# Prepartum Fat Mobilization in Dairy Cows with Equal Body Condition and Its Impact on Health, Behavior, Milk Production and Fertility during Lactation

**DOI:** 10.3390/ani10091478

**Published:** 2020-08-22

**Authors:** Alfredo Rodríguez, Ricardo Mellado, Hedie Bustamante

**Affiliations:** 1 Graduate School, Faculty of Agricultural Sciences, Universidad Austral de Chile, Valdivia 5110566, Chile; alfredo.rodriguez@postgrado.uach.cl (A.R.); ricardo.mellado.reyes@gmail.com (R.M.); 2 Veterinary Clinical Sciences Institute, Faculty of Veterinary Sciences, Universidad Austral de Chile, Valdivia 5110566, Chile

**Keywords:** fat mobilization, inflammation, oxidative stress

## Abstract

**Simple Summary:**

An excess of lipolysis and subsequent increase on non-esterified fatty acids concentrations may impair animal health, welfare, and productivity after calving. In this study, we evaluated the effect of fat mobilization in dairy cows with a recommended body condition score at the beginning of the close-up period on blood indicators of health, incidence of diseases, behavior, milk production, and fertility during postpartum. An increased fat mobilization in dairy cows with an equal body condition score modified the inflammatory and oxidative stress responses during the early postpartum without impairing their health status and fertility. Moreover, behavior and milk production were sensitive indicators that reflected the negative effects of the excess of prepartum fat mobilization through lactation.

**Abstract:**

The objective of this study was to evaluate the effect of two levels of fat mobilization at the close-up period in dairy cows with an equal body condition score (BCS = 3.0) on the circulating concentrations of metabolic, inflammatory, and oxidative stress biomarkers, incidence of diseases, behavior, milk production, and fertility during the postpartum. Late-gestation multiparous Holstein cows (*n* = 59) with a body condition score of 3.0 (5-point scale) were enrolled at the beginning of the close-up period and then were followed during the entire lactation. Cows were retrospectively allocated into two groups: animals with prepartum non-esterified fatty acids concentration over 0.3 mmol/L were categorized as high fat mobilization (HFM) (*n* = 26), and below this threshold as low fat mobilization (LFM) (*n* = 33). Blood samples were collected 21 d before expected calving and once weekly for 3 wk postpartum in order to analyze β-hydroxybutirate, haptoglobin, fibrinogen, total proteins, and malondialdehyde. Health was observed daily for 21 d postpartum. Behavioral data was collected with an accelerometer and milk production and fertility were obtained from the farm records. An increased fat mobilization in dairy cows with equal BCS modified the inflammatory and oxidative stress responses during the early postpartum without impairing their health status and fertility. Moreover, milk production and behavior were markedly affected by excessive prepartum fat mobilization through lactation.

## 1. Introduction

Fat mobilization is a common process in dairy cows that supports the high energy demand during the transition period. This process involves lipolysis, where non-esterified fatty acids (NEFA) are generated from triacylglycerols (TAG) in adipocytes by the conflicting action of several hormone-sensitive lipases. Consequently, NEFA are then released into the bloodstream in order to be used as substrate for milk fat synthesis in the udder and also as an energy source in many tissues [1]. However, between 15 to 20% of all NEFA are either partially oxidized to ketone bodies or re-esterified to TAG in the liver [2]. Thus, several studies indicate that excessive lipolysis and the subsequent increase in NEFA concentrations may predispose to TAG accumulation in the liver, oxidative stress, and to a dysfunctional inflammatory response, with all these events leading to further lipolysis, impaired animal health [3,4], welfare [5,6], and productivity [7,8] after parturition.

The degree of fat mobilization depends on many factors such as breed, level of milk production, parity, and nutrition [9]. However, under similar management conditions, dairy cows show high individual variability [10,11,12,13]. Several studies have associated this variability with the development of insulin resistance (IR) [12,14,15], which is defined as a state whereby a normal concentration of insulin induces a decreased biological response in the insulin-sensitive tissues [16]. Furthermore, IR may interfere with insulin-signaling pathways related to the suppression of adipose tissue lipases, which favor a lipolytic response [17]. This scenario has been widely studied in recent years and current research suggests that IR may be exacerbated by several mechanisms. According to Abuelo et al. [18], oxidative stress and the loss of reduction-oxidation (redox) homeostasis results in the accumulation of oxygen radicals that favor an inflammatory response through the activation of the nuclear factor *NF-kB*. As a result, immune cells increase the expression of pro-inflammatory cytokines, which in turn increase reactive oxygen species (ROS) production and disrupt the insulin-signaling downstream, increasing lipolysis [19].

In order to minimize the effects generated by increased fat mobilization, dairy farms thoroughly monitor body condition score (BCS) during the prepartum [6,20]. The BCS is a routine tool that gives a qualitative assessment of body fatness and allows for the identification of animals with increased risk of suffering postpartum disorders [8,21,22]. Nonetheless, according to LeBlanc [23], the sensitivity and specificity of BCS for predicting postpartum diseases and their consequences may be reduced when used only as a monitoring tool. In fact, animals with a prepartum BCS within the recommended range have a 30% incidence of postpartum health disorders [24]. Therefore, the assessment of animals using an objective indicator of fat mobilization such as NEFA, may help elucidate the mechanisms that trigger lipolysis in cows with a similar BCS. Therefore, the objective of this study was to evaluate the effect of two levels of fat mobilization at the close-up period in dairy cows with an equal body condition score (BCS = 3.0) on the circulating concentrations of metabolic (e.g., β-hydroxybutirate), inflammatory (e.g., haptoglobin, fibrinogen, and total proteins), and oxidative stress (e.g., malondialdehyde) biomarkers, incidence of diseases, behavior, milk production, and fertility during the postpartum.

## 2. Materials and Methods

All procedures described in this study were approved by the Austral University of Chile Animal Ethics Committee (#322/2018).

### 2.1. Animals, Housing, and Diets

This study was carried out between August and December 2017 in a commercial dairy farm located in Los Lagos County, Los Ríos Region, Chile (39°51′00″ S, 72°50′00″ W). Fifty-nine pregnant, multiparous (range: 2–7) Holstein-Friesian cows with a body condition of 3.0 (5-point scale; Edmonson et al. [25]) were enrolled upon entry to the close-up group (approximately 21 d before expected calving date). During this period, cows were housed in a freestall barn and fed with formulated diets (Table 1). Immediately after calving, cows were allocated into the lactating group and managed in a daily rotational grazing system consuming perennial ryegrass (*Lolium perenne*) pasture and supplemented two times a day (4:00 and 14:00 h) with a total mixed ration (TMR) and concentrate according to nutritional requirements for lactating cows (details are shown in Table 1). Cows were milked twice a day at 5:00 and 15:00 h in an 80-point rotary milking parlor (Afimilk Ltd., Kibbutz Afikim, Israel). Cows were maintained during the entire lactation in the same lot and hence were managed under the same conditions.

### 2.2. Group Allocation

Cows were retrospectively allocated into two groups based on prepartum non-steroidal fatty acids concentration (NEFA) based on the threshold previously described by Ospina et al. [27]. Animals with NEFA concentration over 0.3 mmol/L were categorized as cows with high fat mobilization (HFM; *n* = 26) and animals with values below this threshold were categorized as cows with low fat mobilization (LFM; *n* = 33). Differences between groups were statistically tested (Student’s *t*-test, *p* < 0.001).

### 2.3. Blood Sample Collection and Analysis

Blood samples were collected 21 d before the expected calving date, and on wk 1 (between d 3 to 7), wk 2 (between d 8 to 14), and wk 3 (between d 15 to 21) postpartum. Blood samples were collected during the morning (approximately 5 h after feeding), by coccygeal venipuncture, and stored on ice immediately after collection. Plasma and serum were obtained after centrifugation at 3000× *g* for 15 min at room temperature. Samples were then aliquoted in 1.5 mL tubes (Eppendorf, Germany) and frozen at −20 °C until further analysis.

Plasma concentrations of NEFA (Randox, Crumlin, United Kingdom) and Serum concentrations of β-hydroxybutirate (BHB) (Ranbut, Randox, Crumlin, United Kingdom) were measured by enzymatic analysis using an auto-analyzer (Metrolab 2300, Wiener Lab, Rosario, Argentina) and expressed in mmol/L. Plasma concentrations of malondialdehyde (MDA) were measured using the thiobarbituric acid (TBARs) assay, as previously described by Ohkawa et al. [28]. Absorbance was measured at 540 nm with a microplate reader (Tecan GmbH, model Sunrise, Grödig/Salzburg, Austria) and results were expressed in nmol/mL. Serum concentrations of haptoglobin (Hp) were determined photometrically according to the method described by Elson [29] and validated for bovine use [30]. Absorbance was measured at 380 and 405 nm with a spectrophotometer (Thermo scientific evolution 300, Madison, WI, USA) and results were expressed in mg/mL. Plasma fibrinogen (Fb) and total protein (TP) concentrations were determined by refractometry (Atago, Atago co., Washington, DC, USA) according to the heat precipitation method described by Millar et al. [31], and results were expressed in mg/mL.

### 2.4. Health Status and Herd Retention

Health status was assessed daily during the first 21 d postpartum by farm personnel under veterinary supervision in order to identify diseases such as retained placenta, displaced abomasum, clinical mastitis, clinical hypocalcemia, and clinical ketosis [32]. Additionally, metritis diagnosis was performed in order to identify mild and severe cases of this disease [33]. To accomplish this, postpartum body temperature and vaginal discharge were evaluated by a trained veterinarian (AR) once per week during the first 21 d according to the method described in Huzzey et al. [34]. Herd retention was obtained from the farm records and consisted of the number of cows that remained in the herd finishing lactation (305-d). The decision for culling was made by the farm manager, who was blind to the groups, and the researchers did not have influence over the culling decision.

### 2.5. Behavioral Data

Behavioral data was collected during the first 21 d postpartum. Within 2 days after calving, cows were fitted with an accelerometer (AfiTag pedometer plus, Afimilk Ltd. Kibbutz Afikim, Israel) in the right hind limb, which allowed the registration of daily activity (steps/h), lying time (min), and lying bouts (no./d). Lying bout duration (min) was calculated by dividing the lying time by the number of lying bouts on that day.

### 2.6. Milk Production and Reproductive Data

Milk production and reproductive data were obtained from farm records. Milk production was registered daily using an automated system (Afimilk Ltd., Kibbutz Afikim, Israel). Fat, protein, urea, and somatic cell count (SCC) were analyzed individually at 4-wk intervals by the commercial milk-control of the dairy farm (Cooprinsem Ltd., Osorno, Chile). Reproductive data was used to calculate the calving to first service interval (CFSI), defined as the number of days between parturition and the first artificial insemination (AI); calving to conception interval (CCI), defined as the number of days between parturition and the service that results in a pregnancy; conception rate at first service (CRFS), defined as the number of first services that resulted in pregnancy divided by the total number of first services; pregnancy rate (PR), defined as the number of pregnant cows during the current lactation divided by the total number of animals synchronized; and services per conception (SC), defined as the number of AIs required to conceive.

### 2.7. Statistical Analysis

Data were analyzed using generalized linear mixed models by the GENLINMIXED procedure of SPSS (version 25.0, SPSS Inc., IBM, Chicago, IL, USA). Health status was modeled using a binomial distribution with logit-link function including group (HFM/LFM) as the fixed effect and cow as the random effect. Modelling for blood biomarkers, behavior, milk production, and reproduction included the fixed effects of group (HFM and LFM), time (day, week, or month), and their interaction, and the random effect of cow. Additionally, the blood biomarkers model included the prepartum sample as the covariate. Repeated measures over time were modeled with first-order autoregressive and Toeplitz covariance structures, which were selected based on the lowest Akaike information criterion. Nonsignificant terms were eliminated based on Wald statistic backward selection criterion (*p* > 0.15), excluding the main effects, which were forced in the final models. Herd retention data was analyzed using Kaplan-Meier survival curves and differences were determined by the Log-Rank test. Cows culled or dead before 305 d were censored. For all analysis, the significance level was set at *p* < 0.05 and tendencies were identified at 0.05 ≤ *p* < 0.10.

## 3. Results

### 3.1. Blood Biomarkers

Blood biomarker concentrations are presented in Table 2. Overall, fat mobilization was not associated with changes on circulating concentrations of BHB (*p* = 0.70), Fb (*p* = 0.22), or TP (*p* = 0.43). However, MDA and Hp concentrations were higher in the HFM group than in the LFM group (*p* = 0.01 and *p* = 0.03, respectively). All evaluated biomarkers were influenced by time (*p* ≤ 0.01), with the exception of BHB (*p* = 0.45). Similarly, the interaction between group and time was not associated with changes in the concentrations of BHB (*p* > 0.15), MDA (*p* = 0.14), Fb (*p* = 0.10), or TP (*p >* 0.15). Nonetheless, Hp concentration in the HFM group was higher than the LFM group during the first week (0.14 ± 0.04 mg/mL vs. 0.04 ± 0.01 mg/mL; *p* = 0.03) and then decreased during weeks two (0.06 ± 0.01 vs. 0.03 ± 0.01; *p* = 0.06) and three (0.02 ± 0.01 vs. 0.03 ± 0.01; *p* = 0.52). Moreover, despite the fact that group by time interaction for MDA was not significant (*p* = 0.14), the HFM group showed a marked increase in MDA concentration compared with the LFM group during week two (estimated means ± SEM = 16.5 ± 1.3 vs. 12.7 ± 0.7, respectively; *p* < 0.01).

### 3.2. Health Status and Herd Retention

Overall, the fat mobilization level was not associated with changes in the incidence of clinical metritis (HFM = 50% vs. LFM = 48.5%, *p* = 0.91), puerperal metritis (HFM = 7.7% vs. LFM = 9.0%, *p* = 0.87), clinical mastitis (HFM = 3.8 vs. LFM = 0%, *p* = 0.42), displaced abomasum (HFM = 3.8 vs. LFM = 0%, *p* = 0.42), one event of disease (HFM = 57.6 vs. LFM = 57.6%, *p* = 0.99), or multiple events of disease (HFM = 7.7 vs. LFM = 0%, *p* = 0.12). Likewise, herd retention was not influenced by fat mobilization (*p* = 0.75).

### 3.3. Behavior

**Figure 1:**
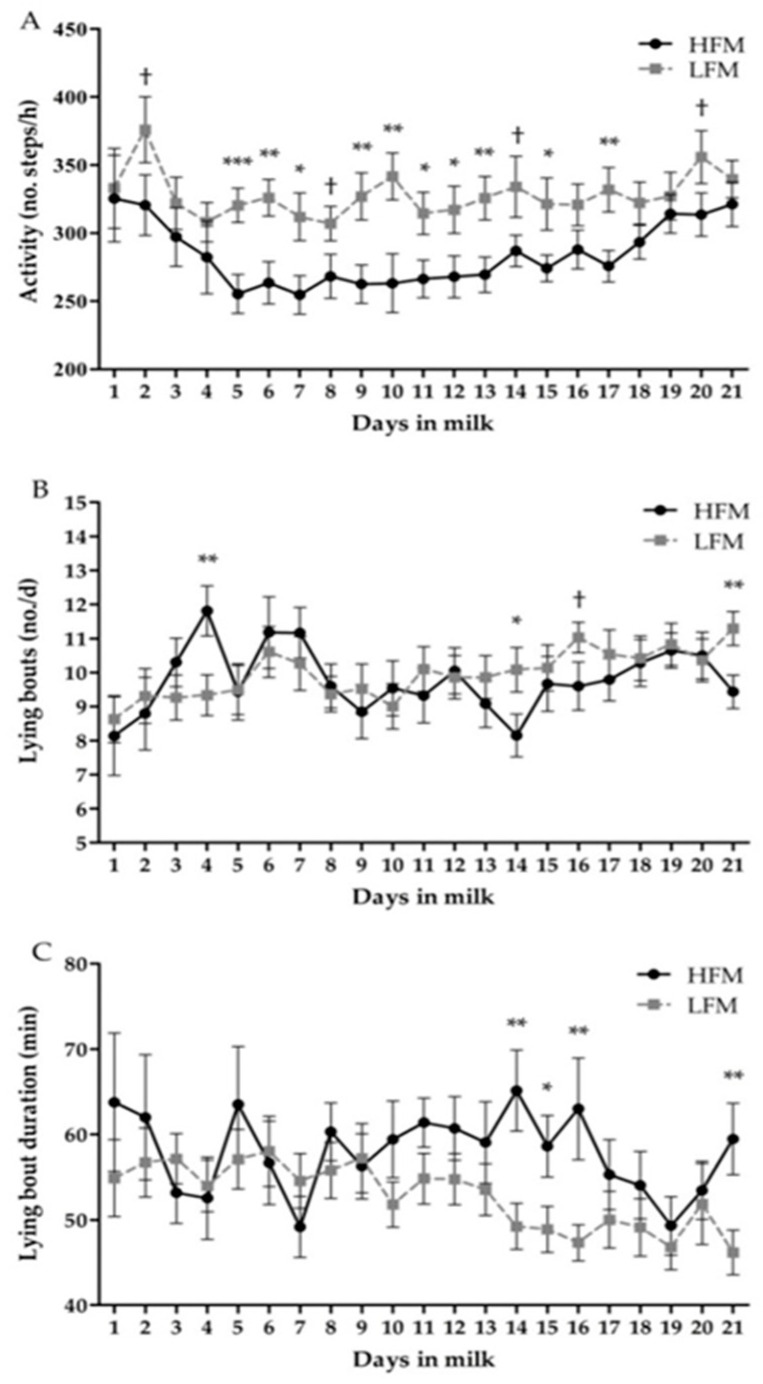
Daily activity (**A**), lying bouts (**B**), and lying bout duration (**C**) in the high fat (HFM) and low fat (LFM) mobilization groups during the first 21 days postpartum. Values are expressed as estimated means ± SEM. * *p* < 0.05; ** *p* < 0.01; † 0.05 ≤ *p* < 0.10. Lying time was excluded due *p* > 0.15.

Daily activity and lying behavior are summarized in Table 3. Overall, cows in the HFM group were less active than cows in the LFM group, reducing their activity by 43.8 ± 14 steps/h (*p* < 0.01) and increasing their lying time by 35.6 min/d (*p* = 0.03). Lying bouts and lying bout duration were not associated with fat mobilization (*p* = 0.67 and *p* = 0.11, respectively). All evaluated variables were influenced by time (*p* < 0.01) and the interaction between group and time was also associated with all variables (*p* < 0.01) with the exception of lying time (*p* > 0.15). Daily activity was decreased in the HFM group at d 2 through d 20 compared with the LFM group (Figure 1A). Similarly, lying bouts in the HFM group were increased on d 3 (2.5 ± 0.9 bouts/d; *p* < 0.01) and then decreased at d 14 (1.9 ± 0.9 bouts/d; *p* = 0.03) and 21 (1.9 ± 0.7 bouts/d; *p* < 0.01) compared with the LFM group (Figure 1B). Moreover, lying bouts duration was higher in the HFM group, increasing at days 14 (15.9 ± 5.5 min/d; *p* < 0.01), 15 (9.7 ± 4.5 min/d; *p* = 0.03), 16 (15.7 ± 6.3 min/d; *p* = 0.01), and 21 (13.3 ± 4.9 min/d; *p* < 0.01) (Figure 1C).

### 3.4. Milk Production and Composition

**Figure 2:**
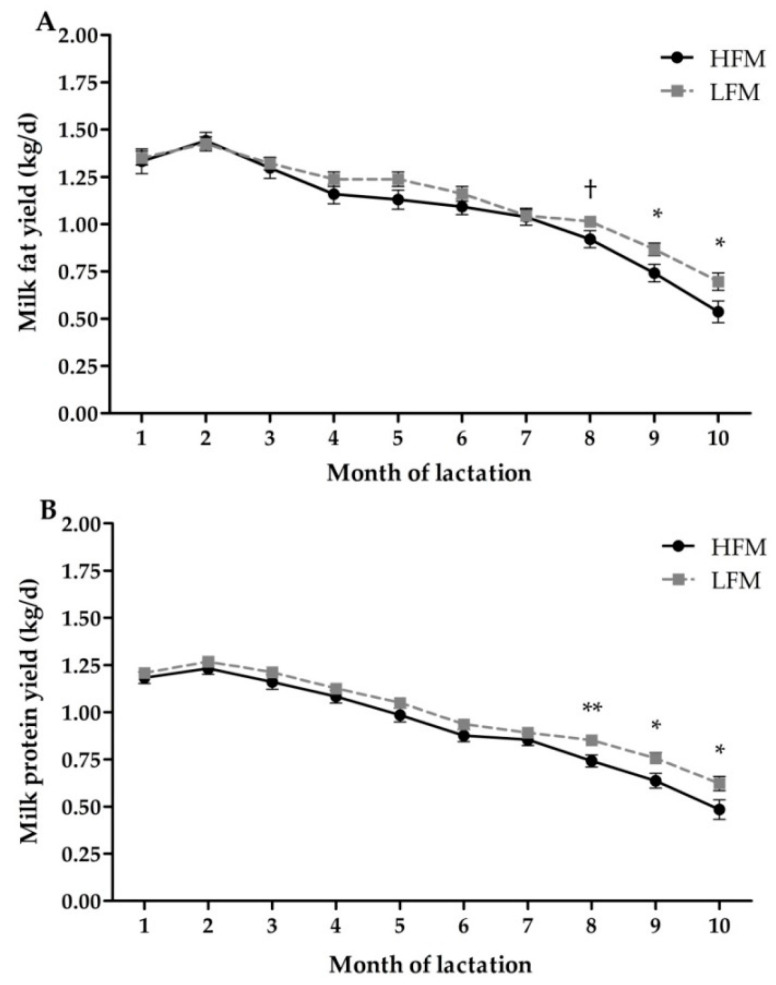
Daily milk fat (**A**) and protein (**B**) yields in high fat (HFM) and low fat (LFM) mobilization groups. * *p* < 0.05; ** *p* <0.01; † 0.05 ≤ *p* < 0.10.

Whole-lactation milk production and composition are summarized in Table 4. Fat mobilization was not associated with changes in milk and fat yield. However, cows in the HFM group produced less protein (0.07 ± 0.03 kg/d; *p* = 0.03) compared with cows in the LFM group. All variables were influenced by time (*p* < 0.01). Similarly, the fat mobilization by time interaction showed that cows in the HFM group produced less milk fat during the ninth (0.125 ± 0.06 kg/d; *p* = 0.03) and tenth (0.159 ± 0.07 kg/d; *p* = 0.03) month of lactation compared to cows in the LFM group (Figure 2A). Moreover, a tendency to reduce the milk protein (*p* = 0.08) was observed in the HFM group compared to the LFM group during the last three months of lactation (average = 122.3 kg/d; Figure 2B), whereas milk yields were unaltered (*p* > 0.15). Milk components were unaffected by fat mobilization groups (all comparisons *p* ≥ 0.35) or by the interaction between fat mobilization groups and time (all comparisons *p* ≥ 0.09).

### 3.5. Reproduction

Fat mobilization was not associated with changes in any reproductive variable evaluated (*p* ≥ 0.3, Table 5).

## 4. Discussion

The objective of this study was to evaluate the effect of two levels of fat mobilization in prepartum dairy cows with an equal body condition score (BCS 3.0) on the concentrations of blood indicators of health, incidence of diseases, behavior, milk production, and fertility during postpartum. In order to a obtain a better understanding about the metabolic and inflammatory processes affected by fat mobilization during the transition period, this study was performed in a commercial dairy farm where cows were managed during the entire lactation under the same conditions. Despite having the same body condition score upon enrolling, dairy cows showed different levels of fat mobilization, with 42% of total cows showing higher NEFA concentrations during the prepartum (HFM = 690.1 ± 73.9 mmol/L vs. LFM = 197.4 ± 10.9 mmol/L; *p* < 0.001).

Increased fat mobilization did not alter plasma concentrations of BHB. Nonetheless, BHB has been previously considered to be biomarker of health status [35]. Ospina et al. [36] indicate that the plasma concentration of NEFA and BHB have a weak correlation, with only 18% of the variability of BHB explained by NEFA. In this study, a similar nutritional regimen was maintained and hence, the results here described for BHB may be related to differences in the gluconeogenic capacity and/or with the availability of other carbon sources different than NEFA, including lactate or ketogenic amino acids [37].

Plasma MDA concentration was higher in the HFM group than in the LFM group. Malondialdehyde is the final product of lipid peroxidation, a process that occurs as a consequence of oxidative stress [18,38]. Although the values here reported are in the middle of the range of those previously reported for dairy cows during the transition period [39,40,41,42,43], they followed a similar pattern, characterized by a significant increase in the first two weeks after calving. These results suggest that both groups experienced limited degrees of oxidative stress, and cows in the HFM group were increasingly exposed to oxidative damage during the second week postpartum.

Serum concentration of Hp was significantly higher in the HFM group, whereas plasma Fb did not differ between groups. Moreover, both biomarkers showed a similar pattern characterized by higher concentrations during the first week that decreased gradually on weeks two and three. However, the observed increase of Hp and Fb was slightly higher than those previously reported for healthy animals [44]. According to Bertoni et al. [45], acute phase proteins such as Hp and Fb are synthesized in the liver in response to the release of pro-inflammatory cytokines, increasing their concentrations in direct relation with the degree of inflammation. This fact would confirm that an increase in fat mobilization may induce various degrees of inflammation, particularly during the first week postpartum. The results here presented are in agreement with previous studies in which dairy cows were grouped according to their prepartum or early postpartum NEFA concentration [12,46]. In both cases, an increase in fat mobilization was associated with increased plasma concentration of acute phase proteins after calving. However, based on previous studies, we observed that some differences could arise when dairy cows were grouped on the basis of BCS. Rafia et al. [47] described that Fb tended to increase after calving in cows with moderate or higher BCS during the prepartum. Similarly, Roche et al. [48] observed a marked increase in plasma Hp after calving in cows with higher BCS, which were offered 100 and 125% of their energy requirements compared to cows with low BCS fed under the same nutritional regimen. Likewise, Montagner et al. [49] observed that dairy cows with a decrease in BCS had an increased inflammatory status around calving compared to those animals that increased their BCS. On the contrary, Roche et al. [50] and Schuh et al. [51] indicate that BCS was not associated with changes in acute phase response during the early postpartum. Taking this into account, the results presented here would confirm that the use of BCS does not assure a success in adaptation through the transition period and it highlights the importance of identifying those animals with high fat mobilization using more sensitive methods.

The changes in oxidative stress and inflammatory markers observed in cows in the HFM group were not associated with the presence of specific diseases. Cows in both groups were similarly affected by a high incidence of health disorders, which did not have an effect on animal culling as explained by herd retention. Moreover, fat mobilization did not have an effect on reproductive tract diseases and fertility. According to Sordillo and Raphael [19], an increase in fat mobilization and a subsequent increase in NEFA concentration may predispose to TAG accumulation in the liver and overproduction of ketone bodies. Furthermore, the increased demand for energy during early lactation increases the production of reactive oxygen species (ROS), leading to oxidative stress and, hence, to hepatic damage [52]. However, current research indicates that NEFA and ROS can also activate receptors present in immune cells (e.g., toll-like receptors, nuclear receptors, and inflammasomes), leading to a chronic inflammatory condition that is responsible for both metabolic and inflammatory diseases during the transition period [53]. Accordingly, we believe that the lack of a relationship between oxidative stress/inflammation and the incidence of disease in cows of the HFM group may reflect a normal status of the mechanisms that regulate disease resolution [17]. These mechanisms are regulated by the biosynthesis of resolving oxylipids, a class of omega 3 polyunsaturated fatty acid (PUFA) -derived lipid mediators, including maresins, protectins, and resolvins [53,54]. An important source of precursors of resolving oxylipids during the transition period is the inclusion of high concentrations of omega 3 in the ration, which may be internalized into membrane phospholipids of macrophages, monocytes, and vascular endothelial cells, favoring an adequate anti-inflammatory response [55]. Unfortunately, in this study, we did not evaluate the fatty acid composition of the ration, however the inclusion of a perennial ryegrass pasture on the postpartum diets could have contributed to increase the total amount of omega 3 available [56].

Assessment of daily activity patterns showed that cows in the HFM group were less active and spent more time lying down than those cows in the LFM group following calving. Although no specific information studying the role of prepartum NEFA concentration on postpartum behavior is currently available, some evidence indicates that behavior is modified in animals that suffer from disorders or diseases causing stress and pain [57,58,59,60]. As previously mentioned, the incidence of disease did not differ between groups, which could confirm that fat mobilization plays an important role influencing daily activity during the postpartum, regardless of the presence of disease. This finding partially agrees with the negative correlation between plasma concentration of NEFA and daily activity described during the postpartum by Adewuyi et al. [61]. Moreover, the behavioral changes here described in cows with increased fat mobilization could be associated with a higher degree of oxidative stress and inflammation. According to Bertoni et al. [45], this pro-inflammatory status during the transition period may influence and alter normal patterns of hepatic synthesis of proteins, partitioning of nutrients, reproductive activity, body temperature, and induce anorexia and lethargy, which could alter feeding or social behaviors [62].

Although prepartum fat mobilization decreased milk fat and protein yields during lactation, we believe that those changes were mostly influenced by numerical differences in milk yield (HFM = 1.65 ± 1.04 kg/d less milk). This result demonstrates that prepartum fat mobilization had a cumulative effect through lactation, which was possibly associated with the inflammatory status observed during the postpartum. This assumption is supported by previous studies, in which cows treated with sodium salicylate after calving had an increase in milk yield during lactation [63,64]. Nonetheless, the mechanisms by which inflammation affects milk production during lactation remain unclear. A recent study by Kvidera et al. [65] suggest that differences in milk production could be related to an increase in glucose requirements needed to maintain a more active immune system. We believe that cows in the HFM group possibly redirected disposable glucose previously destined for milk synthesis towards their immune system as an evolutionary response [66]. Nonetheless, research is needed in order to confirm this assumption.

## 5. Conclusions

This study showed that increased fat mobilization in dairy cows with an equal BCS modified the inflammatory and oxidative stress response during the early postpartum without impairing their health status and fertility. Furthermore, increased levels of prepartum fat mobilization markedly affected behavior and milk production through lactation.

## Figures and Tables

**Table 1:** Ingredients and chemical analysis (DM basis) of the ration offered through the study.

Items	Diets	
	Close-Up Dry Cows	Lactating Cows
Ingredients, % of DM		
Wheat Straw	21.78	3.59
High-moisture corn	-	6.93
Soybean meal	7.26	5.02
Steam-flaked corn	-	6.31
Canola meal	14.36	3.90
Triticale grain ground	-	9.93
Corn gluten meal	5.57	-
Corn silage	44.55	12.23
Pasture ^1^	-	47.62
Molasses beet	-	2.14
Mycotoxin binder ^2^	0.08	0.04
Magnesium sulphate	0.57	-
Magnesium oxide	-	0.32
Sodium bicarbonate	-	0.80
Mineral mix podological	-	1.20
Anionic salts ^3^	5.02	-
Rumen buffer ^4^	0.81	-
Chemical analysis		
NE_L_, Mcal/kg of DM	1.35	1.84
Crude protein, % DM	17.82	20.33
Crude fat, % DM	2.93	3.72
aNDFom, % DM	40.38	30.93
ADF, % DM	27.18	16.49
Calcium, % DM	0.76	0.54
Phosphorus, % DM	0.37	0.45
Magnesium, % DM	0.53	0.47

^1^ For more details, see Sube et al. [26]. ^2^ Mycosorb A+ (Alltech, Lexington, KY, USA). ^3^ Soychlor (West Central Cooperative, Ralston, IA, USA). ^4^ Acid Buf (AB Vista, Marlborough, Wiltshire, England).

**Table 2:** Concentrations of blood biomarkers related to energy balance (β-hydroxybutirate, BHB), oxidative stress (malondialdehyde, MDA), and inflammation (haptoglobin (Hp), fibrinogen (Fb), and total proteins (TP)) in high fat (HFM) and low fat (LFM) mobilization groups.

Item ^1^	Groups			*p*-Value		
	HFM	LFM	SEM	Group	Time	G × T
BHB, mmol/L	0.67	0.65	0.05	0.70	0.45	>0.15
MDA, nmol/mL	13.92	12.11	0.72	0.01	<0.01	0.14
Haptoglobin, mg/mL	0.07	0.02	0.02	0.03	<0.01	0.01
Fibrinogen, mg/mL	3.19	3.53	0.28	0.22	<0.01	0.10
Total proteins, mg/mL	76.26	75.33	1.17	0.43	<0.01	>0.15

^1^ Values are expressed as estimated means and SEM.

**Table 3:** Daily activity and lying behavior in the high fat (HFM) and low fat (LFM) mobilization groups.

Items ^1^	Groups			*p*-Value		
	HFM	LFM	SEM	Groups	Time	G × T
Activity (no. steps/h)	283.9	327.8	14.06	<0.01	<0.01	<0.01
Lying time (min/d)	516.9	481.3	16.50	0.03	<0.01	>0.15
Lying bouts (no./d)	9.8	10.0	0.44	0.67	<0.01	<0.01
Lying bout duration (min)	57.9	52.8	3.14	0.11	<0.01	<0.01

^1^ Values are expressed as estimated means and SEM.

**Table 4:** Milk production and composition in high fat (HFM) or low fat (LFM) mobilization groups.

Item ^1^	Groups			*p*-Value		
	HFM	LFM	SEM	Group	Time	G × T
Milk yield (kg/d)	27.96	29.61	1.04	0.11	<0.01	>0.15
Protein (%)	3.51	3.49	0.06	0.83	<0.01	0.12
Fat (%)	4.11	3.98	0.14	0.35	<0.01	>0.15
Protein yield (kg/d)	0.92	0.99	0.03	0.03	<0.01	0.08
Fat yield (kg/d)	1.07	1.13	0.04	0.14	<0.01	0.04
Milk urea (mg/dL)	37.07	38.01	1.25	0.45	<0.01	0.14
Somatic cell linear score ^2^	2.22	2.02	0.35	0.59	<0.01	0.09

^1^ Values are expressed as estimated means and SEM. ^2^ Calculated using the following equation: log_2_(SCC/100) + 3. SCC = somatic cell count.

**Table 5:** Reproductive variables in high fat (HFM) and low fat (LFM) mobilization groups.

Variables ^1^	Groups		*p*-Value
	HFM	LFM	
Conception rate at first service, %	52 (10)	50 (8.6)	0.88
Pregnancy rate, %	88.1 (5.4)	88 (5.5)	0.66
Calving to first service interval, d	72 (2.3)	72.6 (1.9)	0.84
Calving to conception interval, d	98.2 (7.9)	104.9 (7.9)	0.55
Services per conception, no.	1.8 (0.2)	2.2 (0.2)	0.30

^1^ Values are expressed as estimated means and SEM.

## References

[1] Contreras G.A., Sordillo L. (2011). Lipid mobilization and inflammatory responses during the transition period of dairy cows. Comp. Immunol. Microbiol. Infect. Dis..

[2] Drackley J.K., Andersen J.B., Sejrsen K., Hvelplund T., Nielsen M.O. (2006). Splanchnic metabolism of long-chain fatty acids in ruminants. Ruminant Physiology: Digestion, Metabolism and Impact of Nutrition on Gene Expression, Immunology and Stress.

[3] Huzzey J., Nydam D., Grant R., Overton T. (2011). Associations of prepartum plasma cortisol, haptoglobin, fecal cortisol metabolites, and nonesterified fatty acids with postpartum health status in Holstein dairy cows. J. Dairy Sci..

[4] Esposito G., Irons P.C., Webb E.C., Chapwanya A. (2014). Interactions between negative energy balance, metabolic diseases, uterine health and immune response in transition dairy cows. Anim. Reprod. Sci..

[5] Loor J., Bertoni G., Hosseini A., Roche J.R., Trevisi E. (2013). Functional welfare—Using biochemical and molecular technologies to understand better the welfare state of peripartal dairy cattle. Anim. Prod. Sci..

[6] Roche J.R., Kay J.K., Friggens N.C., Loor J., Berry D. (2013). Assessing and Managing Body Condition Score for the Prevention of Metabolic Disease in Dairy Cows. Vet. Clin. N. Am. Food Anim. Pract..

[7] Ospina P., Nydam D., Stokol T., Overton T. (2010). Associations of elevated nonesterified fatty acids and β-hydroxybutyrate concentrations with early lactation reproductive performance and milk production in transition dairy cattle in the northeastern United States. J. Dairy Sci..

[8] Barletta R., Filho M.M., Carvalho P., Del Valle T., Netto A., Rennó F., Mingoti R., Gandra J., Mourão G., Fricke P. (2017). Association of changes among body condition score during the transition period with NEFA and BHBA concentrations, milk production, fertility, and health of Holstein cows. Theriogenology.

[9] Nielsen H., Friggens N., Løvendahl P., Jensen J., Ingvartsen K. (2003). Influence of breed, parity, and stage of lactation on lactational performance and relationship between body fatness and live weight. Livest. Prod. Sci..

[10] Kessel S., Stroehl M., Meyer H.H.D., Hiss S., Sauerwein H., Schwarz F.J., Bruckmaier R.M. (2008). Individual variability in physiological adaptation to metabolic stress during early lactation in dairy cows kept under equal conditions. J. Anim. Sci..

[11] Weber C., Hametner C., Tuchscherer A., Losand B., Kanitz E., Otten W., Singh S., Bruckmaier R., Becker F., Kanitz W. (2013). Variation in fat mobilization during early lactation differently affects feed intake, body condition, and lipid and glucose metabolism in high-yielding dairy cows. J. Dairy Sci..

[12] Humer E., Khol-Parisini A., Gruber L., Wittek T., Aschenbach J.R., Zebeli Q. (2016). Metabolic adaptation and reticuloruminal pH in periparturient dairy cows experiencing different lipolysis early postpartum. Animal.

[13] Zachut M., Moallem U. (2017). Consistent magnitude of postpartum body weight loss within cows across lactations and the relation to reproductive performance. J. Dairy Sci..

[14] Bell A.W. (1995). Regulation of organic nutrient metabolism during transition from late pregnancy to early lactation. J. Anim. Sci..

[15] De Koster J., Opsomer G. (2013). Insulin Resistance in Dairy Cows. Vet. Clin. N. Am. Food Anim. Pract..

[16] Kahn C.R. (1978). Insulin resistance, insulin insensitivity, and insulin unresponsiveness: A necessary distinction. Metabolism.

[17] Sordillo L.M., Mavangira V. (2014). The nexus between nutrient metabolism, oxidative stress and inflammation in transition cows. Anim. Prod. Sci..

[18] Abuelo A., Hernandez J., Benedito J.L., Castillo C., Rodríguez C.C. (2014). The importance of the oxidative status of dairy cattle in the periparturient period: Revisiting antioxidant supplementation. J. Anim. Physiol. Anim. Nutr..

[19] Sordillo L., Raphael W. (2013). Significance of Metabolic Stress, Lipid Mobilization, and Inflammation on Transition Cow Disorders. Vet. Clin. N. Am. Food Anim. Pract..

[20] Bewley J., Schutz M. (2008). An Interdisciplinary Review of Body Condition Scoring for Dairy Cattle. Prof. Anim. Sci..

[21] Pires J.A., Delavaud C., Faulconnier Y., Pomiès D., Chilliard Y. (2013). Effects of body condition score at calving on indicators of fat and protein mobilization of periparturient Holstein-Friesian cows. J. Dairy Sci..

[22] Gärtner T., Gernand E., Gottschalk J., Donat K. (2019). Relationships between body condition, body condition loss, and serum metabolites during the transition period in primiparous and multiparous cows. J. Dairy Sci..

[23] Leblanc S.J. (2010). Monitoring Metabolic Health of Dairy Cattle in the Transition Period. J. Reprod. Dev..

[24] Contreras L., Ryan C., Overton T. (2004). Effects of Dry Cow Grouping Strategy and Prepartum Body Condition Score on Performance and Health of Transition Dairy Cows. J. Dairy Sci..

[25] Edmonson A., Lean I., Weaver L., Farver T., Webster G. (1989). A Body Condition Scoring Chart for Holstein Dairy Cows. J. Dairy Sci..

[26] Sube A., Aguirre C., Dec D., Balocchi O., Alonso M.F. (2016). Yield and quality of Lolium perenne L. pastures under irrigation in the Southern Zone of Chile. Agro Sur.

[27] Ospina P., Nydam D., Stokol T., Overton T. (2010). Evaluation of nonesterified fatty acids and β-hydroxybutyrate in transition dairy cattle in the northeastern United States: Critical thresholds for prediction of clinical diseases. J. Dairy Sci..

[28] Ohkawa H., Ohishi N., Yagi K. (1979). Assay for lipid peroxides in animal tissues by thiobarbituric acid reaction. Anal. Biochem..

[29] Elson E.C. (1974). Quantitative Determination of Serum Haptoglobin: A Simple and Rapid Method. Am. J. Clin. Pathol..

[30] Hirvonen J., Pyörälä S., Jousimies-Somer H. (1996). Acute phase response in heifers with experimentally induced mastitis. J. Dairy Res..

[31] Millar H.R., Simpson J.G., Stalker A.L. (1971). An evaluation of the heat precipitation method for plasma fibrinogen estimation. J. Clin. Pathol..

[32] Kelton D.F., Lissemore K.D., Martin R.E. (1998). Recommendations for Recording and Calculating the Incidence of Selected Clinical Diseases of Dairy Cattle. J. Dairy Sci..

[33] Sheldon I., Owens S.E. (2017). Postpartum uterine infection and endometritis in dairy cattle. Anim. Reprod..

[34] Huzzey J., Veira D., Weary D., Von Keyserlingk M.A. (2007). Prepartum Behavior and Dry Matter Intake Identify Dairy Cows at Risk for Metritis. J. Dairy Sci..

[35] Ospina P., Nydam D., Stokol T., Overton T. (2010). Association between the proportion of sampled transition cows with increased nonesterified fatty acids and β-hydroxybutyrate and disease incidence, pregnancy rate, and milk production at the herd level. J. Dairy Sci..

[36] Ospina P.A., McArt J.A., Overton T.R., Stokol T., Nydam D.V. (2013). Using Nonesterified Fatty Acids and β-Hydroxybutyrate Concentrations During the Transition Period for Herd-Level Monitoring of Increased Risk of Disease and Decreased Reproductive and Milking Performance. Vet. Clin. N. Am. Food Anim. Pract..

[37] McCarthy M., Mann S., Nydam D., Overton T., McArt J. (2015). Short communication: Concentrations of nonesterified fatty acids and β-hydroxybutyrate in dairy cows are not well correlated during the transition period. J. Dairy Sci..

[38] Sordillo L., Aitken S.L. (2009). Impact of oxidative stress on the health and immune function of dairy cattle. Vet. Immunol. Immunopathol..

[39] Castillo C., Hernandez J., Bravo A., López-Alonso M., Pereira V., Benedito J. (2005). Oxidative status during late pregnancy and early lactation in dairy cows. Vet. J..

[40] Castillo C., Hernandez J., Valverde I., Pereira V., Sotillo J., López-Alonso M., Benedito J., Rodríguez C.C. (2006). Plasma malonaldehyde (MDA) and total antioxidant status (TAS) during lactation in dairy cows. Res. Vet. Sci..

[41] Sharma N., Singh N.K., Singh O.P., Pandey V., Verma P.K. (2011). Oxidative Stress and Antioxidant Status during Transition Period in Dairy Cows. Asian-Australas. J. Anim. Sci..

[42] Konvičná J., Vargová M., Paulíková I., Kovác G., Kostecká Z. (2015). Oxidative stress and antioxidant status in dairy cows during prepartal and postpartal periods. Acta Vet. Brno.

[43] Colakoglu H.E., Yazlik M.O., Kaya U., Çolakoğlu E.Ç., Kurt S., Öz B., Bayramoglu R., Vural M.R., Küplülü Ş. (2017). MDA and GSH-Px activity in transition dairy cows under seasonal variations and their relationship with reproductive performance. J. Vet. Res..

[44] Ceciliani F., Cerón J.J., E Silva F.C., Sauerwein H. (2012). Acute phase proteins in ruminants. J. Proteom..

[45] Bertoni G., Trevisi E., Han X., Bionaz M. (2008). Effects of Inflammatory Conditions on Liver Activity in Puerperium Period and Consequences for Performance in Dairy Cows. J. Dairy Sci..

[46] Hiss S., Weinkauf C., Hachenberg S., Sauerwein H. (2009). Short communication: Relationship between metabolic status and the milk concentrations of haptoglobin and lactoferrin in dairy cows during early lactation. J. Dairy Sci..

[47] Rafia S., Taghipour-Bazargani T., Khaki Z., Bokaie S., Tabrizi S.S. (2011). Effect of body condition score on dynamics of hemogram in periparturient Holstein cows. Comp. Haematol. Int..

[48] Roche J.R., Meier S., Heiser A., Mitchell M.D., Walker C.G., Crookenden M.A., Riboni M.V., Loor J., Kay J. (2015). Effects of precalving body condition score and prepartum feeding level on production, reproduction, and health parameters in pasture-based transition dairy cows. J. Dairy Sci..

[49] Montagner P., Krause A.R.T., Schwegler E., Weschenfelder M.M., Maffi A.S., Xavier E.G., Schneider A., Pereira R.A., Jacometo C.B., Schmitt E. (2017). Relationship between pre-partum body condition score changes, acute phase proteins and energy metabolism markers during the peripartum period in dairy cows. Ital. J. Anim. Sci..

[50] Roche J.R., Macdonald K., Schütz K., Matthews L., Verkerk G., Meier S., Loor J., Rogers A., McGowan J., Morgan S. (2013). Calving body condition score affects indicators of health in grazing dairy cows. J. Dairy Sci..

[51] Schuh K., Sadri H., Häussler S., Webb L.A., Urh C., Wagner M., Koch C., Frahm J., Dänicke S., Dusel G. (2019). Comparison of performance and metabolism from late pregnancy to early lactation in dairy cows with elevated v. normal body condition at dry-off. Animal.

[52] Du X., Chen L., Huang D., Peng Z., Zhao C., Zhang Y., Zhu Y., Wang Z., Li X., Liu G. (2017). Elevated Apoptosis in the Liver of Dairy Cows with Ketosis. Cell. Physiol. Biochem..

[53] Bradford B.J., Swartz T.H. (2020). Review: Following the smoke signals: Inflammatory signaling in metabolic homeostasis and homeorhesis in dairy cattle. Animal.

[54] Mavangira V., Sordillo L.M. (2018). Role of lipid mediators in the regulation of oxidative stress and inflammatory responses in dairy cattle. Res. Vet. Sci..

[55] Raphael W., Sordillo L.M. (2013). Dietary Polyunsaturated Fatty Acids and Inflammation: The Role of Phospholipid Biosynthesis. Int. J. Mol. Sci..

[56] Palladino R.A., O’Donovan M., Kennedy E., Murphy J.J., Boland T.M., Kenny D.A. (2009). Fatty acid composition and nutritive value of twelve cultivars of perennial ryegrass. Grass Forage Sci..

[57] Sepúlveda-Varas P., Weary D.M., Von Keyserlingk M.A. (2014). Lying behavior and postpartum health status in grazing dairy cows. J. Dairy Sci..

[58] Kaufman E., Leblanc S.J., McBride B., Duffield T., Devries T.J. (2016). Short communication: Association of lying behavior and subclinical ketosis in transition dairy cows. J. Dairy Sci..

[59] Barragan A., Piñeiro J., Schuenemann G., Rajala-Schultz P.J., Sanders D., Lakritz J., Bas S. (2018). Assessment of daily activity patterns and biomarkers of pain, inflammation, and stress in lactating dairy cows diagnosed with clinical metritis. J. Dairy Sci..

[60] Piñeiro J., Menichetti B., Barragan A., Relling A., Weiss W., Bas S., Schuenemann G. (2019). Associations of pre- and postpartum lying time with metabolic, inflammation, and health status of lactating dairy cows. J. Dairy Sci..

[61] Adewuyi A., Roelofs J., Gruys E., Toussaint M., Van Eerdenburg F. (2006). Relationship of Plasma Nonesterified Fatty Acids and Walking Activity in Postpartum Dairy Cows. J. Dairy Sci..

[62] Munksgaard L., Jensen M.B., Pedersen L.J., Hansen S.W., Matthews L. (2005). Quantifying behavioural priorities—Effects of time constraints on behaviour of dairy cows, Bos taurus. Appl. Anim. Behav. Sci..

[63] Farney J., Mamedova L., Coetzee J.F., Minton J., Hollis L., Bradford B.J. (2013). Sodium salicylate treatment in early lactation increases whole-lactation milk and milk fat yield in mature dairy cows. J. Dairy Sci..

[64] Carpenter A., Ylioja C., Vargas C., Mamedova L., Mendonça L., Coetzee J.F., Hollis L., Gehring R., Bradford B.J. (2016). Hot topic: Early postpartum treatment of commercial dairy cows with nonsteroidal antiinflammatory drugs increases whole-lactation milk yield. J. Dairy Sci..

[65] Kvidera S., Horst E., Abuajamieh M., Mayorga E., Fernandez M.V.S., Baumgard L.H. (2017). Glucose requirements of an activated immune system in lactating Holstein cows. J. Dairy Sci..

[66] Ballou M.A. (2012). Growth and development symposium: Inflammation: Role in the etiology and pathophysiology of clinical mastitis in dairy cows1. J. Anim. Sci..

